# Death of Sir David Barry, with the Appearances on Dissection

**Published:** 1836-01-01

**Authors:** 


### Obituary.

### Sir David Barry.

We have to announce, with sincere regret, the demise of a highly talented and respected member of our profession, since the last number of our Journal went to press! Those who were personally acquainted with Sir David Barry—and he had an extensive circle of acquaintance—will long remember the varied acquirements, (professional and general,)—the great facilities of communicating information—the ready talent for discussion of any subject, whether in the offensive or defensive—the wit, the humour, and the satire, which were always at his command! Yet those who were foiled by the strength of his arguments, or the ingenuity of his reasonings, never felt hurt by the wounds which he inflicted, so tempered were they by the perfect good humour that accompanied them, and the gentlemanly terms in which his strictures or criticisms were conveyed. He was the life of every medical society which he attended—and of every private party in which he appeared. His public career is well known. He distinguished himself, as an army-surgeon, in the Peninsular campaign—his researches on the circulation and on poison, in Paris, are before the public;—his mission to St. Petersburgh, by order of Government, to investigate the nature and march of cholera—his subsequent appointment at the Cholera Board, &c. are too recent to require particular notice. Although a contagionist, in respect to the late epidemic, his views were rational and moderate, as compared with some of his brethren. When the ORIGINAL BOARD, composed *chiefly* of learned collegiate Thebans, not one of whom had ever seen a case of the disease, fell to pieces under its own weight, Dr. Barry, in conjunction with Sir William Russell, issued a code of instructions something in accordance with the 19th century, and not on the model of the BLACK DEATH and PLAGUE DOCTRINES, promulgated by the preceding collegiate Board! He ordained no "*cordons sanitaires*"—no buckets to be suspended from windows for receiving provisions—no private fortresses to be erected, to insulate one neighbour from another! Sir David probably thought, in accordance with many other conscientious individuals, that it was most safe to err (if it was an error) on the side of contagion, striking off all vexatious quarantines and prohibitions of intercourse in this great commercial nation. The good effects of this liberal view of the epidemic have been abundantly manifested. This country has experienced a comparative immunity from cholera, while other less enlightened nations have suffered more from the blind restrictions imposed on the inhabitants than the disease could have possibly inflicted!

But this by the way. Sir David Barry having finished his labours in this department, and being honoured with knighthood, was subsequently employed, under Government, as a Commissioner of inquiry respecting the state of his own unfortunate country—IRELAND.

He had only recently returned from this mission, when his life approached its close! On Tuesday, the 3d of November, he dined out, and ate some pears, nuts, and other matters of difficult digestion. He was in good health and spirits. On Wednesday morning he was on his way to the City, when he was suddenly seized, under the Arcade of the Opera House, in the Haymarket, with a violent pain in his stomach and loins—faintness—chilliness—and collapse. He was carried into a shop, and sent over for Dr. Johnson immediately. Dr. J. being out, Mr. H. Johnson went to his assistance, and found him in the abovementioned condition. His pulse was small, and he considered himself as dying. He was anxious to take an emetic, which was not agreed to. He was conveyed home in a hackney-coach, and put to bed. Dr. Johnson saw him in half an hour. He was still a little chilly, but heat was returning to the surface, and the pulse was beginning to develop itself. He attributed his sufferings to the indigestible substances he had eaten, and begged to have an emetic. Dr. J. would not agree to this, considering that the matters eaten the preceding day had now passed into the bowels, and ought to be carried off by stool. He instantly gave the patient one grain of opium and four of calomel, to be followed, in two hours, by a warm purgative mixture, taken in divided doses till the bowels were freely opened. Dr. J. returned in an hour, and found Sir David much relieved. The pain was diminished, the pulse natural, the skin warm. He had no cough, no oppression at the chest, no tenderness on pressure in any part of the abdomen. The aperient mixture was commenced at four o'clock, and Mr. M'Intyre administered an enema which was retained. Dr. Copland joined Dr. J. and Mr. M. at half-past eight o'clock. Three doses of the medicine had been taken without effect. A large enema of warm water, salt, and oil, was now thrown up, and the bowels were copiously evacuated by two or three motions. The pulse was now about 84, and rather full. He was a little sick, and brought up the last dose of medicine, without straining. He now felt much better, and began to lose the impression that he was dying. The medical attendants staid with him till half-past ten, leaving an anodyne with him, in case hypercatharsis (of which he had a dread) should come on. He remained quiet till one o'clock, when he got up to the night-chair, and instantly expired!

The body was examined by Mr. H. J. Johnson, and Mr. C. Johnson, in presence of Dr. Copland, Mr. M'Intyre, Dr. Johnson, and others. The layer of fat on the abdomen was of remarkable thickness. Nothing unhealthy presented itself in the abdomen. The ribs were ossified. On cutting into the right side, an immense gush of clear serum came out, and impressed us with the idea that he died of hydrothorax! On prosecuting the dissection, however, we found large coagula of blood, the whole effusion or hæmorrhage amounting to full five pints. The left ventricle of the heart was greatly hypertrophied, being full an inch in thickness. The valves were sound. The aorta was dilated from the arch downwards, and an aneurism was found near where the vessel passes through the diaphragm, larger than an orange. This had burst into the posterior mediastinum, and the blood ultimately made its way through the pleura into the right cavity of the chest, where it suddenly destroyed life.

Thus the whole mystery was cleared up. The aneurism had burst while Sir David was walking down the Haymarket; but the hæmorrhage producing faintness, was confined to the posterior mediastinum, causing the pains which he experienced during the next twelve hours. But while on the night-chair, at one o'clock, the grand gush took place into the chest, producing instantaneous death. It is fortunate that the emetic was not given, as there is almost a moral certainty that the patient would have died during its operation. And even if an examination had been permitted, there would still have been great doubt whether the aneurism had not burst in consequence of the vomiting!

This is another instance of the little inconvenience, which fatal organic diseases sometimes produce till near the final termination. Sir David Barry dined out on Tuesday, and was the gayest of the gay—at a time when there was hypertrophy of the heart, dilatation of the aorta, and thoracic aneurism! This highly-gifted physician was in the 56th year of his age, and in full vigour of intellect. About four or five years ago, he requested Dr. Johnson to examine his chest with the stethoscope, as he suspected he had some affection of the heart. Dr. J. could not then detect any disease, but advised Sir David, as he was very much inclined to corpulency, to adopt a spare system of diet by way of precaution. There is reason to believe that this advice was not followed, as Sir David was fond of good eating and drinking, though he never went to excess in either.

